# Open reduction versus closed reduction percutaneous pinning for the treatment of song type V lateral humeral condyle fractures in children. A short term report

**DOI:** 10.3389/fped.2025.1701100

**Published:** 2025-12-10

**Authors:** Jianwen Fang, Dahui Wang, Lujian Tan, Yunbo Peng, Yueqiang Mo

**Affiliations:** 1 Department of Orthopaedics Surgery, Xiamen Children’s Hospital (Children’s Hospital of Fudan University Xiamen Hospital), Xiamen, Fujian, China; 2 Department of Orthopaedics Surgery, Children’s Hospital of Fudan University, Shanghai, China

**Keywords:** lateral humeral condyle fracture, closed reduction, open reduction, efficacy, elbow function

## Abstract

**Objective:**

To evaluate the efficacy of closed reduction percutaneous pinning (CRPP) in the treatment of Song V lateral humeral condyle fractures in children.

**Methods:**

Medical records of pediatric patients who underwent surgical treatment for lateral humeral condyle fractures at our institution from July 2018 to August 2024 were retrospectively reviewed. Inclusion criteria: (1). Age ≤14 years; (2). Time from injury to surgery ≤3 days; (3). Song V lateral humeral condyle fracture. Exclusion criteria: (1). Open fracture or concomitant fractures; (2). Associated neurovascular injury; (3). Pathological fracture or concomitant metabolic diseases; (4). Incomplete clinical or radiological data; (5). History of previous surgery on the ipsilateral elbow. Patients meeting the criteria were divided into two groups based on surgical approach: open reduction (*n* = 43) and closed reduction (*n* = 46). Gender, age, affected side, intraoperative blood loss, and operative duration were recorded. Elbow function was assessed using the Mayo Elbow Performance Score (MEPS) at the one-year postoperative follow-up. Overgrowth of the lateral condyle was evaluated on anteroposterior radiographs by the presence of a prominent lateral spur; overgrowth was defined as a final interepicondylar width (IEW)/initial IEW ratio >1.1.

**Results:**

The mean age in the closed reduction group was 4.87 ± 1.55 years, compared to 4.53 ± 1.57 years in the open reduction group, showing no statistically significant difference (*t* = 1.0126, *P* = 0.314). There were also no significant differences between the two groups in terms of gender distribution (*χ²* = 2.715, *P* = 0.099) or affected side (left/right) (*χ²* = 0.01, *P* = 0.914). The closed reduction group demonstrated significantly less intraoperative blood loss (1.02 ± 0.15 mL vs. 3.77 ± 3.22 mL; *U* = 240.5, *P* < 0.001) and shorter operative duration (52.96 ± 19.52 min vs. 91.84 ± 30.16 min; *U* = 275.0, *P* < 0.001). At the one-year follow-up, no significant difference was found in MEPS (99.78 ± 1.02 vs. 99.53 ± 1.45; *U* = 841.0, *P* = 0.180). Evaluation of lateral condyle overgrowth also showed no significant difference between the groups (*χ*^2^ = 2.46, *P* = 0.12).

**Conclusion:**

Closed reduction percutaneous pinning fixation for Song V lateral humeral condyle fractures achieves surgical outcomes comparable to open reduction. However, CRPP offers the advantages of minimal scarring and significantly shorter operative time.

## Introduction

Lateral humeral condyle fractures (LHCFs) are the second most common elbow fractures in children after supracondylar fractures, accounting for 12%–20% of pediatric elbow fractures (1–3). The treatment strategy depends on the degree of displacement and stability (4–7). Non-displaced fractures can be managed with immobilization in a cast (7, 8); severely displaced fractures (>2 mm), especially those with fragment rotation, typically require surgical intervention (5, 6, 9).

For cases requiring surgery, traditional teaching holds that open reduction and internal fixation (ORIF) is the preferred treatment to achieve congruent articular reduction and adequate fracture alignment (7, 8). Recent studies, however, suggest that closed reduction percutaneous pinning (CRPP) may be a more appropriate option (5, 10, 11). Existing data indicate no significant difference in complication rates or prognosis between CRPP and ORIF (5, 10, 12).

Historically, ORIF has been considered the gold standard for displaced and unstable LHCFs, aiming for anatomical reduction and stable fixation (5, 8, 9). However, ORIF carries potential complications such as infection, unsightly scarring, and disruption of the blood supply to the fracture fragment (5, 9). In recent years, CRPP, as a less traumatic alternative, has gained increasing attention. Despite advantages like smaller incisions, less blood loss, and shorter operative times, the efficacy and safety of CRPP, particularly for completely displaced and rotated Song V fractures, remain controversial (5, 10, 13).

This retrospective analysis investigates the clinical outcomes of open reduction vs. closed reduction with Kirschner wire fixation for the treatment of Song V LHCFs.

## Materials and methods

### Clinical data

This study was approved by the hospital ethics committee (Approval No. 20250801-2), and informed consent was obtained from the guardians of all participating children. All methods were conducted in accordance with the Declaration of Helsinki.

Medical records of pediatric patients who underwent surgical treatment for LHCFs at our center from July 2018 to August 2024 were collected. Inclusion criteria: 1. Age ≤14 years; 2. Time from injury to surgery ≤3 days; 3. Diagnosis of Song V lateral humeral condyle fracture. Exclusion criteria: (1). Open fracture or concomitant fractures at other sites; (2). Associated neurovascular injury; (3). Pathological fracture or concomitant metabolic diseases; (4). Incomplete clinical or radiological data; (5). History of previous surgery on the ipsilateral elbow.

Among a total of 90 eligible patients with Song V LHCFs, one was excluded after conversion to open reduction due to failure to achieve an acceptable reduction (displacement <2 mm). Consequently, 46 cases (33 male, 13 female) were included in the closed reduction group and 43 (24 male, 19 female) in the open reduction group. See Table 1. Age, affected side, intraoperative blood loss, and operative duration were recorded. Following the initiation of a continuous quality improvement project in January 2021 focused on closed reduction for Song Type V LHCFs, the cases enrolled in this retrospective study are categorized as follows: those from 2021 onward underwent closed reduction, while those from 2018 to 2020 were treated with conventional open reduction.

**Table 1:** Comparison of baseline characteristics of two groups.

Characteristic	Closed reduction	Open reduction	*t/χ²*	*P*
M/F (n)	33/13	24/19	2.72	0.099
Age [(χ ± *s*) yrs]	4.87 ± 1.55	4.53 ± 1.57	1.01	0.314
Left/Right (*n*)	23/23	22/21	0.01	0.914

Yrs, years.

Elbow function was assessed using the Mayo Elbow Performance Score (MEPS) at the one-year postoperative follow-up. The maximum distance between the medial and lateral epicondyles of the distal humerus (Interepicondylar Width, IEW) was measured on initial and final follow-up anteroposterior radiographs. Lateral condyle spurring (overgrowth) was defined as a final IEW/initial IEW ratio >1.1 (14). Baseline characteristics of the two groups are presented in Table 1.

### Main surgical procedure

**Figure 1:**
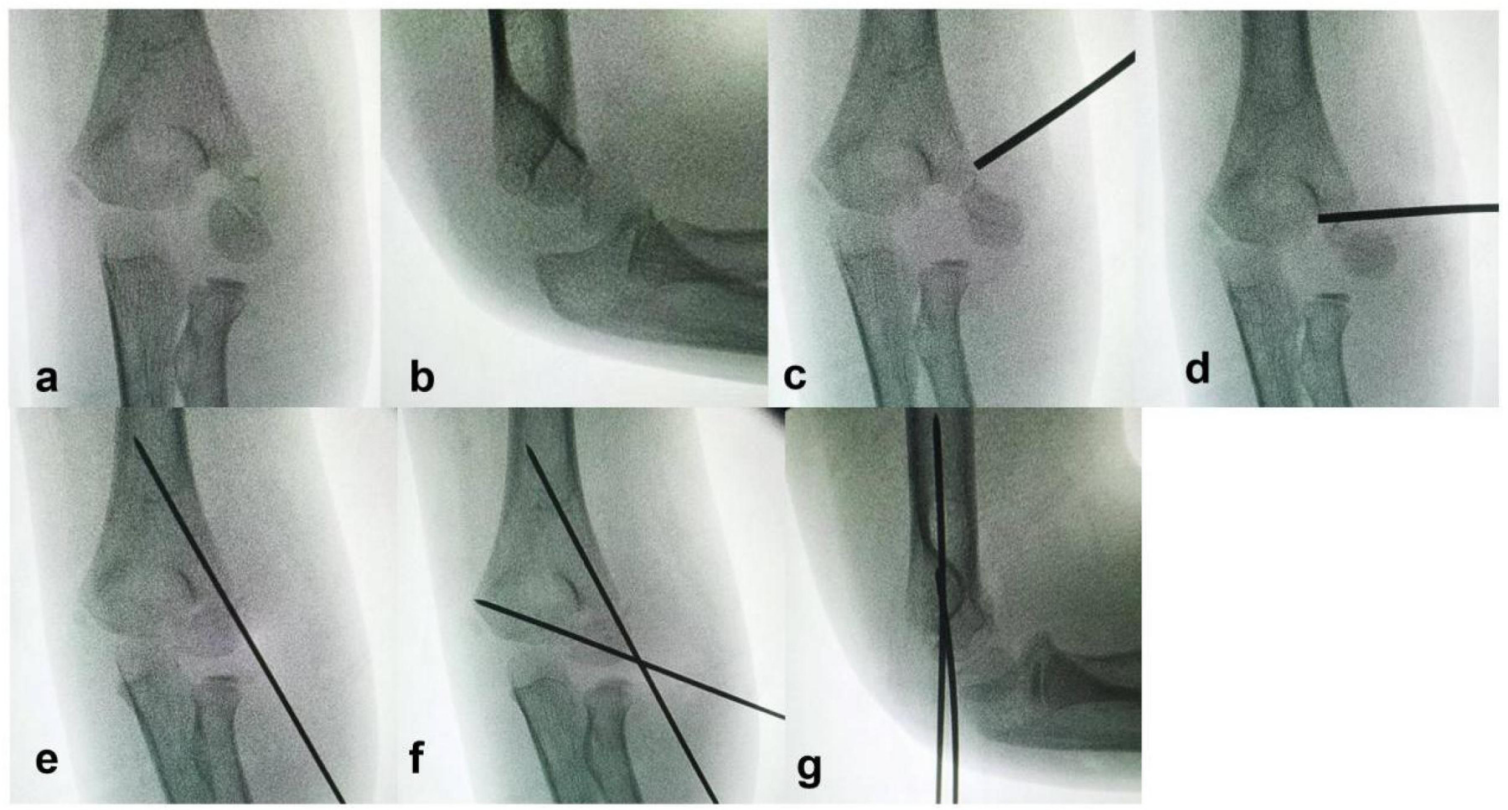
Intraoperative fluoroscopic images of lever reduction and Kirschner wire fixation for a Song V lateral humeral condyle fracture. **(a,b)** Displaced fracture under fluoroscopy. **(c)** Kirschner wire tail placed between the rotated fragment and the proximal humeral metaphysis. **(d)** Lever reduction correcting the rotational displacement. **(e)** After manual correction of lateral displacement, one Kirschner wire fixes the fracture. **(f,g)** Compression along the first wire reduces the fracture gap, and the second wire is inserted.

Closed Reduction Percutaneous Pinning (CRPP) (Figure 1): Under fluoroscopic guidance (C-arm), the position of the rotated fracture fragment was located. A 2.0 mm Kirschner wire was inserted at the medial edge of the rotated fragment. The wire tail was used as a lever to correct the rotational displacement, converting the Song V fracture to a Song IV or III pattern. Manual reduction was then performed to correct lateral displacement by applying compression along the fracture line. A 1.5 mm Kirschner wire was placed perpendicular to the fracture line for posterolateral fixation of the fragment. Further manual reduction was applied along the direction of the first K-wire until intraoperative fluoroscopy (AP, lateral, and oblique views) confirmed a fracture gap of less than 2 mm on all views, and a second 1.5 mm Kirschner wire was inserted for cross fixation. Both wires penetrated the opposite cortex.

Open Reduction Internal Fixation (ORIF): A lateral Kocher approach was used with a 3–4 cm incision. The interval between the brachioradialis and triceps brachii muscles was developed. Dissection proceeded distally along the hematoma, through the disrupted joint capsule to reach the fracture site, taking care to preserve the periosteal blood supply. The fracture ends were exposed, and any hematoma or interposed soft tissue was debrided. The articular surface of the distal fragment was anatomically reduced. Fixation was achieved with two diverging Kirschner wires penetrating the opposite cortex.

### Postoperative management and follow-up

Kirschner wires were bent 90° at the skin level and cut. The ends were covered with sterile gauze to prevent pin tract infection and skin penetration. Wires were removed upon confirmation of fracture healing, which was defined by radiographic evidence of continuous callus formation and the disappearance of the fracture line, coupled with the absence of tenderness or pain upon axial loading at the fracture site. Patients were instructed in rehabilitation exercises.

All patients were followed for 1 year via outpatient clinic visits or video calls. Clinical and radiographic assessments were performed every 2 weeks postoperatively until fracture union and pin removal. Loose casts were replaced, and pin sites dress were changed. Further assessments occurred at 3 months, 6 months, and 1 year postoperatively. Clinical evaluation used the MEPS, assessing range of motion, pain, stability, and functional ability. Radiographic evaluation assessed union, nonunion, fragment absorption/necrosis, and spur formation. Complications were defined as: unplanned secondary surgery, osteomyelitis, nonunion/malunion, avascular necrosis, cubitus varus/valgus, and joint stiffness.

### Statistical analysis

Statistical analysis was performed using SPSS 23.0. Continuous data are presented as mean ± standard deviation (*χ* ± s) and compared using independent samples *t*-tests. Categorical data are presented as percentages or rates and compared using Chi-square (*χ*^2^) or Mann–Whitney *U*-tests. A *P*-value < 0.05 was considered statistically significant.

## Results

Intraoperative blood loss was significantly lower in the closed reduction group (1.02 ± 0.15 mL) compared to the open reduction group (3.77 ± 3.22 mL; *U* = 240.5, *P* < 0.001). Operative duration was significantly shorter in the closed reduction group (52.96 ± 19.52 min) than in the open reduction group (91.84 ± 30.16 min; *U* = 275.0, *P* < 0.001). At the one-year follow-up, MEPS scores (Closed reduction group: 99.78 ± 1.02 vs. Open reduction group: 99.53 ± 1.45; *U* = 841.0, *P* = 0.180) showed no significant difference in functional outcome. Asymptomatic lateral spur formation was observed in 42 cases: 18 (39.13%) in the closed reduction group and 24 (55.81%) in the open reduction group, showing no statistically significant difference (*χ*^2^ = 2.46, *P* = 0.120). See Table 2.

**Table 2:** Comparison of intraoperative parameters and postoperative function between groups.

Parameter	Closed reduction (*n* = 46)	Open reduction (*n* = 43)	*U/χ* ^2^	*P*
Intraoperative blood loss(mL)	1.02 ± 0.15	3.77 ± 3.22	240.5	<0.001
Operation duration(min)	52.96 ± 19.52	91.84 ± 30.16	275.0	<0.001
MEPS	99.78 ± 1.02	99.53 ± 1.45	841.0	0.180
Lateral spur	39.13%	55.81%	2.46	0.120

MEPS, mayo elbow performance score.

All fractures achieved radiographic union within 6 weeks postoperatively, at which point casts were removed and K-wires were extracted. No severe complications occurred, such as nonunion, deformities, or osteomyelitis (Table 3). No patient had elbow flexion/extension limitation exceeding 15°. No significant pin site irritation or granuloma formation was observed in either group. The open reduction group had no cases of wound infection.

**Table 3:** Comparison of significant complications between groups.

Group	Nonunion	Malunion	Avascular necrosis	Cubitus varus	Cubitus valgus	Joint stiffness
Group CRRP	0/46	0/46	0/46	0/46	0/46	0/46
Group ORIF	0/43	0/43	0/43	0/43	0/43	0/43

CRRP, closed reduction percutaneous pinning; ORIF, open reduction internal fixation.

**Figure 2:**
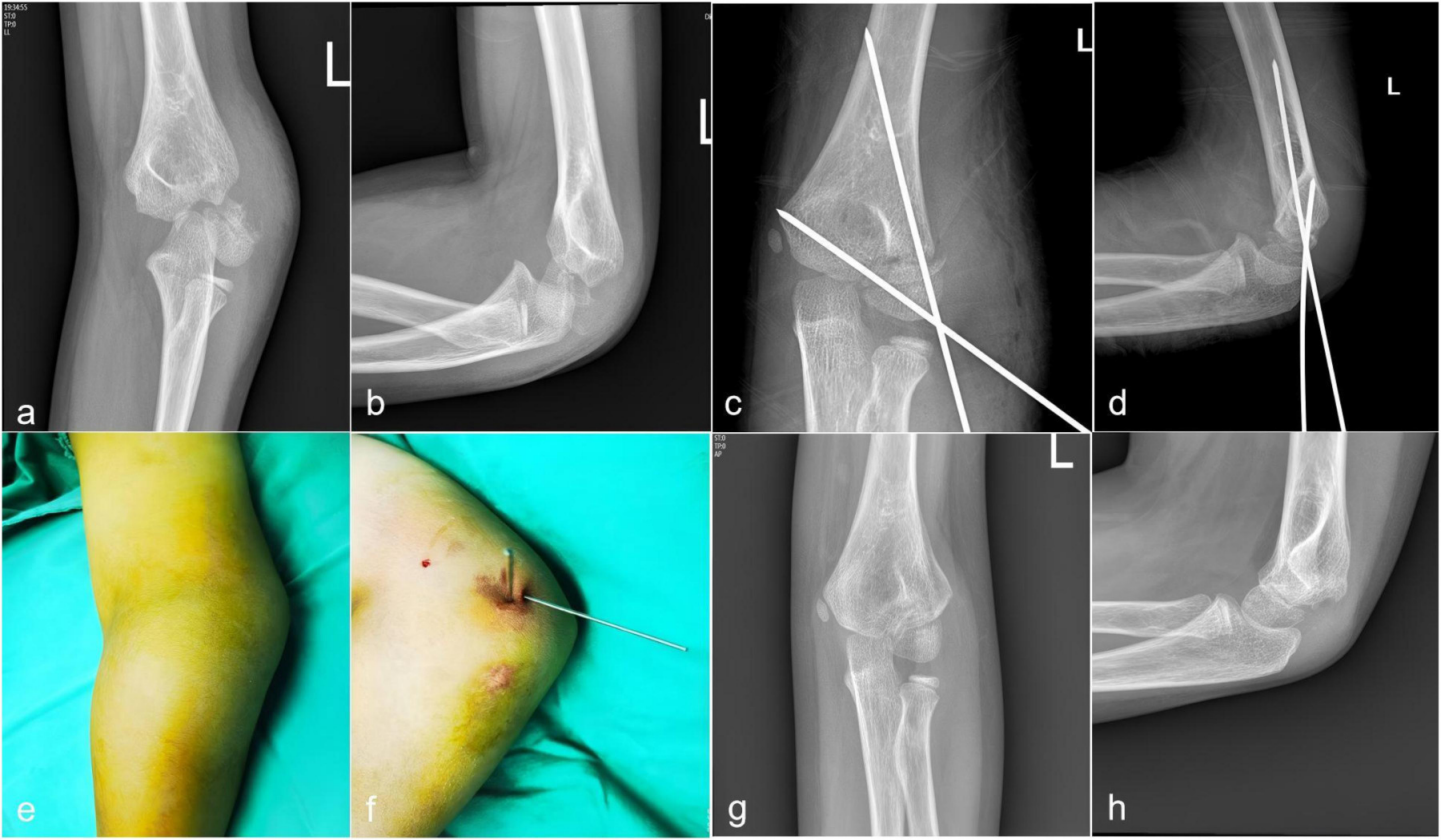
Sequential imaging and clinical appearance of a representative case of Song V lateral humeral condyle fracture treated with closed reduction percutaneous pinning. **(a,b)** Preoperative anteroposterior and lateral radiographs showing complete displacement and rotation of the lateral condylar fragment. **(c,d)** Intraoperative radiographs after successful closed reduction and Kirschner wire placement. **(e,f)** Clinical photographs of the elbow following percutaneous pinning, demonstrating minimal surgical wound. **(g,h)** Follow-up AP radiograph at 12 months postoperatively, showing complete fracture union without evidence of avascular necrosis or significant deformity.

Figure 2 illustrated the sequential imaging and clinical appearance of a representative case of Song V lateral humeral condyle fracture that was successfully treated with closed reduction percutaneous pinning and achieved a final MEPS of 100 points.

## Discussion

Pediatric lateral humeral condylar fractures (LHCFs) are common elbow injuries, and the choice of treatment strategy is crucial for the prognosis of affected children. This study aimed to compare the clinical efficacy of open reduction and internal fixation (ORIF) with closed reduction and percutaneous pinning (CRPP) in the treatment of Song V LHCFs in children. Song V fractures, characterized by complete displacement and rotation, have traditionally been considered complex cases requiring open reduction to achieve satisfactory anatomical reduction (4, 13). However, with the deepening of minimally invasive concepts and advancements in imaging techniques, CRPP has gained increasing popularity in the treatment of pediatric fractures (12, 15–17).

This study demonstrated that the CRPP group exhibited significant advantages over the ORIF group in terms of operative time (52.96 ± 19.52 min vs. 91.84 ± 30.16 min, *P* < 0.001). Regarding intraoperative blood loss, while the CRPP group demonstrates an advantage over open reduction statistically (1.02 ± 0.15 mL vs. 3.77 ± 3.22 mL, *P* < 0.001), the approximately 3 mL difference lacks significant clinical implications. These findings are consistent with numerous previous studies (5, 10, 17, 18), indicating that CRPP, as a minimally invasive surgical approach, can effectively reduce surgical trauma and lower perioperative risks for pediatric patients (10, 15). Xie et al. highlighted that for pediatric LHCFs with displacement greater than 4 mm, CRPP offered advantages over ORIF in terms of operative time, hospital stay, and complication rates (10). This aligns with the growing body of evidence supporting the benefits of less invasive techniques in pediatric orthopedics.

Despite the clear advantages of CRPP in terms of surgical invasiveness, its success rate for complex fractures has always been a focal point of concern. A meta-analysis conducted by Meng C et al. compare the effectiveness of CRPP vs. ORIF to treat the pediatric humeral lateral condylar fracture concluded no significantly different between two groups and find that CRPP offered the benefit of eliminating unaesthetic scar (15). Xu X et al. compared the outcomes of these two fixation techniques concluded that CRPP may result in a higher rate of operative failure but has been found to significantly reduce the occurrence of unsightly scars. Both CRPP and ORPP showed similar levels of postoperative functional satisfaction, with no statistical difference in other complications (5). Our study specifically focused on Song V fractures, a subtype typically associated with severe displacement and rotation of the fracture fragment (11). Conventional wisdom has long held that for such fractures, open reduction is an indispensable means to ensure anatomical reduction (13, 19, 20). However, the successful implementation of CRPP in our study, coupled with the observation that the postoperative Mayo Elbow Performance Score (MEPS) in the CRPP group was not significantly different from that in the ORIF group (99.78 ± 1.02 vs. 99.53 ± 1.45, *P* = 0.180), suggests that under specific conditions, CRPP can also achieve excellent functional outcomes for Song V fractures.

Overgrowth of the lateral condyle is a common complication following pediatric LHCFs, and its pathogenesis is complex, potentially involving factors such as fracture severity, hematoma stimulation, and subperiosteal new bone formation (14, 21). The study by Pribaz JR et al. revealed that overgrowth of the lateral condyle correlates with initial displacement and that overgrowth has more to do with the initial energy and displacement of the fracture rather than the operation itself (14). Our study also found no statistically significant difference in the incidence of overgrowth of the lateral condyle between the two groups (CRPP group: 18 cases, 39.13%; ORIF group: 24 cases, 55.81%).

While our study successfully utilized fluoroscopy to confirm a fracture gap of less than 2 mm, the limitations of plain radiography in visualizing the cartilaginous articular surface are well-documented (22). Intraoperative arthrography has been advocated as a valuable tool to delineate the articular surface, thereby guiding the reduction method and ensuring its adequacy (22–24). This is particularly relevant for minimally displaced fractures, as relying solely on radiographic displacement measurements is insufficient (23). While arthrography provides a more accurate assessment, it is an invasive procedure. Recent studies have explored ultrasound as a non-invasive alternative, showing high consistency with arthrography (24). Therefore, while not universally essential for all cases, we would recommend considering intraoperative arthrography as a supplementary tool to confirm anatomical articular surface reduction, particularly in situations where fluoroscopic assessment is equivocal, thereby enhancing the precision of the surgical intervention.

This study has several limitations that warrant consideration. Firstly, its retrospective design inherently introduces potential biases, including selection bias and confounding factors, which may affect the generalizability of the findings. Secondly, while the MEPS scores indicated comparable functional recovery between the two groups, a detailed comparative analysis of specific complication types, such as nonunion, avascular necrosis, and cubitus varus/valgus deformities, was not performed. This omission limits a comprehensive understanding of the safety profiles of the different surgical approaches. Furthermore, the follow-up period, though sufficient for short-term outcomes, may not be long enough to capture all long-term complications or growth disturbances associated with pediatric lateral humeral condylar fractures. Future prospective studies with larger sample sizes, longer follow-up durations, and more granular reporting of complications are needed to provide more robust evidence and further refine treatment guidelines.

In conclusion, our study contributes to the growing body of literature supporting the efficacy of CRPP for pediatric LHCFs, even for complex Song V fractures. The observed benefits in terms of reduced operative time, coupled with comparable short-term functional outcomes, underscore the value of a minimally invasive approach. Future research should focus on long-term follow-up studies, comprehensive comparative analyses of all potential complications. This will help to further refine treatment algorithms and optimize outcomes for children with LHCFs.

## Data Availability

The original contributions presented in the study are included in the article/Supplementary Material, further inquiries can be directed to the corresponding author.
